# ﻿A new cryptic species in the *Thelodermarhododiscus* complex (Anura, Rhacophoridae) from China–Vietnam border regions

**DOI:** 10.3897/zookeys.1099.80390

**Published:** 2022-05-03

**Authors:** Lingyun Du, Jian Wang, Shuo Liu, Guohua Yu

**Affiliations:** 1 Key Laboratory of Ecology of Rare and Endangered Species and Environmental Protection (Guangxi Normal University), Ministry of Education, Guilin 541004, China; 2 Guangxi Key Laboratory of Rare and Endangered Animal Ecology, College of Life Science, Guangxi Normal University, Guilin 541004, China; 3 Ministry of Education Key Laboratory for Ecology of Tropical Islands & Key Laboratory of Tropical Animal and Plant Ecology of Hainan Province, College of Life Sciences, Hainan Normal University, Haikou 571158, China; 4 College of Biological and Agricultural Sciences, Honghe University, Mengzi 661199, China; 5 Kunming Natural History Museum of Zoology, Kunming Institute of Zoology, Chinese Academy of Sciences, Kunming 650223, China

**Keywords:** 16S rRNA, COI, southern Yunnan, *Thelodermahekouense* sp. nov.

## Abstract

We describe a new species of *Theloderma* from southern Yunnan, China and northern Vietnam based on morphological and molecular evidence. *Thelodermahekouense***sp. nov.**, which had been recorded as *T.rhododiscus*, is the sister to *T.rhododiscus*. The new species differs genetically from *T.rhododiscus* by 4.2% and 10.7% in 16S rRNA and COI genes, respectively, and it can be morphologically distinguished from *T.rhododiscus* by having more densely spaced white warts on the dorsal surface, red subarticular tubercles, red metacarpal tubercles, a red metatarsal tubercle, and black dorsal and ventral surfaces in preservative. Currently the new species is only known from the China–Vietnam border regions of Yunnan and Ha Giang, while *T.rhododiscus* has a wide distributional range in China including Guangxi, Guangdong, Hunan, Fujian, Jiangxi, and presumably Guizhou and eastern Yunnan. Including the new species, there are currently 10 *Theloderma* species in China and seven *Theloderma* species in Yunnan, where more species will probably be found.

## ﻿Introduction

*Theloderma* Tschudi, a genus of the family Rhacophoridae, occurs in southern and eastern areas of Asia and currently contains 26 species (Frost 2021), of which nine are recognized from China and seven are known from Yunnan including *T.albopunctatum* (Liu & Hu), *T.baibungense* (Jiang, Fei & Huang), *T.bicolor* (Bourret), *T.gordoni* Taylor, *T.moloch* (Annandale), *T.pyaukkya* Dever, and *T.rhododiscus* (Liu & Hu) (Du et al. 2020).

**Figure 1.**
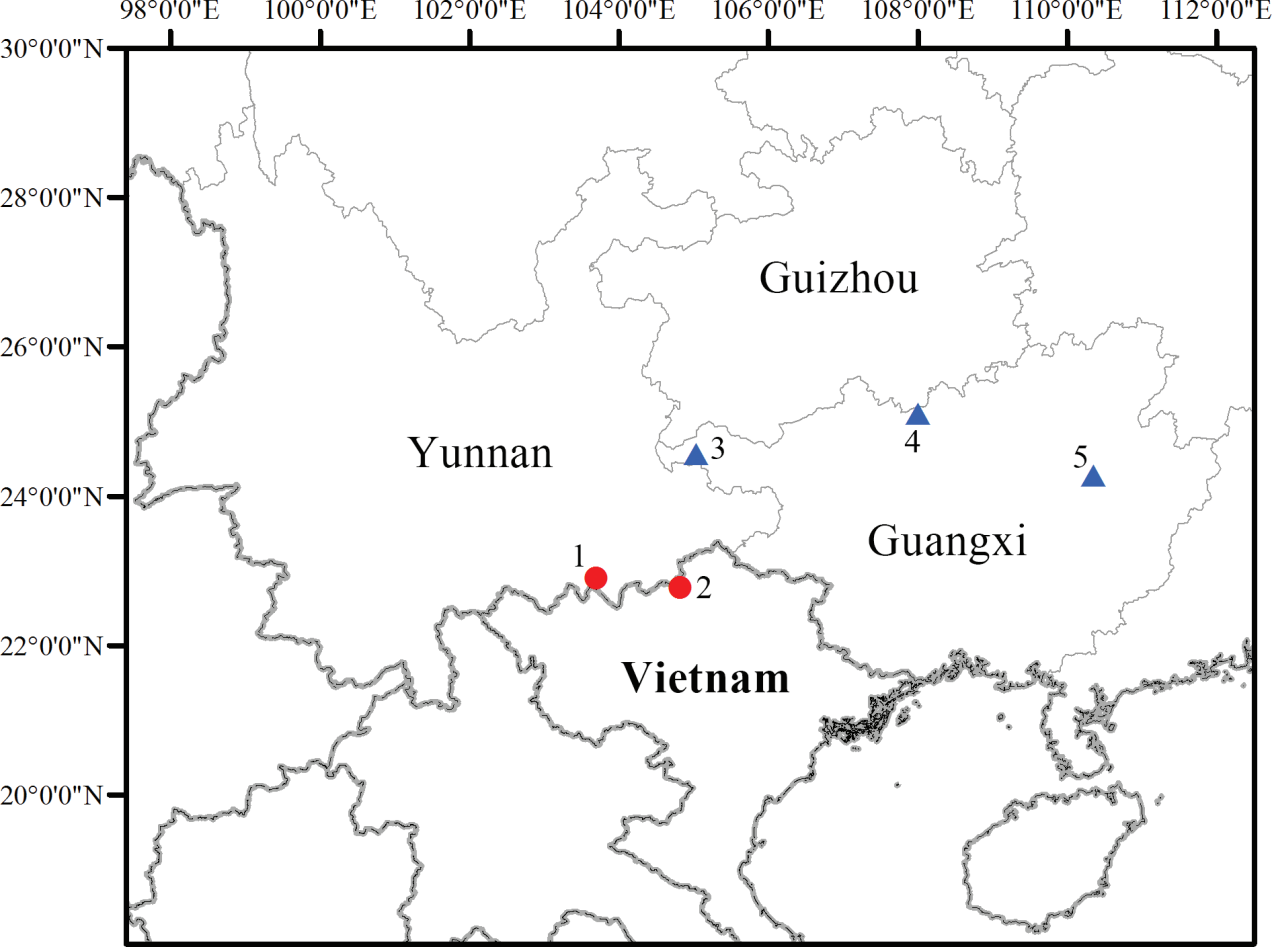
Map showing the collection sites of *T.hekouense* sp. nov. (circle) and *T.rhododiscus* (triangle) in this study **1** Hekou (type locality of the new species) **2** Ha Giang **3** Longlin **4** Huanjiang **5** Jinxiu (type locality of *T.rhododiscus*)

*Thelodermarhododiscus* was originally described from Mt Dayao, Guangxi, China in 1962 (Liu and Hu 1962; Fig. 1) and now is widely recorded from Fujian, Jiangxi, Hunan, Guangdong, Yunnan (Fei et al. 2012; Hou et al. 2017a, 2017b; Zeng et al. 2017), and northern Vietnam (Bain and Nguyen 2004). It was characterized by fingers and toes with orange-red disks, dorsal surface tea-brown, and a dorsum covered with white tubercles interweaved as a network (Liu and Hu 1962).

Numerous studies have shown that widely recorded amphibian species might actually be composed of multiple cryptic species (e.g., Lyu et al. 2019; Yu et al. 2019a, 2019b). Although Zeng et al. (2017) confirmed that the records of *T.rhododiscus* from Guangdong (Mt Nankun) and Jiangxi (Mts Jiulian and Sanbai) are conspecific with *T.rhododiscus* from the type locality based on morphological and molecular evidence, records of *T.rhododiscus* from other places need further confirmation from both morphological and molecular perspectives. Our earlier phylogenetic analysis of *Theloderma* (Hou et al. 2017b) showed that the clade consisting of populations from Yunnan and northern Vietnam is separated from the clade consisting of the topotypes with a relative large genetic divergence, which indicates that more studies are needed to test whether the records of *T.rhododiscus* from Yunnan and Vietnam belong to *T.rhododiscus* or not.

In this study, we compared the *T.rhododiscus* specimens from Yunnan with the topotypes of this species from both morphological and molecular perspectives. Our results supported that the records of *T.rhododiscus* from Yunnan and northern Vietnam warrant distinct taxonomic recognition. Additionally, we confirmed two new distribution sites of *T.rhododiscus* in northwestern (Longlin County) and northern Guangxi (Huanjiang County).

## ﻿Materials and methods

### ﻿Sampling

Specimens were collected by Guohua Yu during fieldwork in Jinxiu and Longlin counties, Guangxi, China in April and June of 2020, by Jian Wang during fieldwork in Hekou County, Yunnan, China in May and September 2020 and 2021, and by Shuo Liu during field surveys in Huanjiang County, Guangxi in September 2019. Specimens were fixed and then stored in 75% ethanol. Liver tissues were preserved in 99% ethanol. All specimens were deposited at Guangxi Normal University (**GXNU**).

### ﻿Morphology

Morphometric data were taken using digital calipers to the nearest 0.1 mm. Morphological terminology follows Yu et al. (2019a). Measurements include: snout–vent length (SVL, from tip of snout to vent); head length (HL, from tip of snout to rear of jaws); head width (HW, width of head at its widest point); snout length (SL, from tip of snout to anterior border of eye); internarial distance (IND, distance between nares); interorbital distance (IOD, minimum distance between upper eyelids); upper eyelid width (UEW, maximum width of upper eyelid); eye diameter (ED, diameter of exposed portion of eyeball); tympanum diameter (TD, the greater of tympanum vertical and horizontal diameters); forearm and hand length (FHL, from elbow to tip of third finger); tibia length (TL, distance from knee to heel); foot length (FL, from proximal end of inner metatarsal tubercle to tip of fourth toe); length of foot and tarsus (TFL, from tibiotarsal joint to tip of fourth toe). Comparative morphological data of other *Theloderma* species were taken from their original descriptions or redescriptions (Taylor 1962; Stuart and Heatwole 2004; Orlov and Ho 2005; Orlov et al. 2006; McLeod and Ahmad 2007; Rowley et al. 2011; Poyarkov et al. 2015, 2018; Nguyen et al. 2016; Sivongxay et al. 2016; Dever 2017; Du et al. 2020).

### ﻿Molecular phylogenetic analyses

Total genomic DNA was extracted from liver tissues. Tissue samples were digested using proteinase K, and subsequently purified following a standard phenol/chloroform isolation and ethanol precipitation. Sequences of 16S rRNA (16S) and cytochrome oxidase subunit I (COI) genes were amplified using the primers and experimental protocols of Du et al. (2020). Sequencing was conducted directly using the corresponding PCR primers. All new sequences were deposited in GenBank under accession numbers OL843957–OL843967 and OL843972–OL843982 (Table 1). Available homologous sequences of members of *Theloderma* were obtained from GenBank (Table 1). *Buergeriaoxycephala*, *Liuixalushainanus*, *Gracixalusjinxiuensis*, and *Nyxtixaluspictus* were selected as hierarchical outgroups according to Yu et al. (2009) and Du et al. (2020).

**Table 1.** Samples used in molecular analyses of this study.

Species	Voucher number	Locality	16s	COI
*Buergeriaoxycephala*	MVZ 230425	Hainan, China	KU244359	KU244459
*Liuixalushainanus*	LJT V15	Hainan, China	KC465826	–
*Gracixalusjinxiuensis*	KIZ 061210YP	Guangxi, China	EU215525	–
*Nyctixaluspictus*	KUHE 53517	Malaysia	LC012863	–
*Thelodermaalbopunctatum*	VNMN JR2887	Vinh Phuc, Vietnam	KU244375	KU244431
*Thelodermalaeve*	NAP01644	Lam Dong, Vietnam	KT461907	–
*Thelodermaleporosum*	LJT W46	Malaysia	KC465841	–
*Thelodermapalliatum*	NAP02516	Lam Dong, Vietnam	KT461903	–
*Thelodermavietnamense*	AMS R174047	Mondol Kiri, Cambodia	JN688171	KU244460
*Thelodermastellatum*	Stel1	Chanthaburi, Thailand	KT461918	–
*Thelodermatruongsonense*	VNMN 4402	Khanh Hoa, Vietnam	LC012847	–
*Thelodermaryabovi*	VNMN 3924	Kon Tum, Vietnam	LC012860	–
*Thelodermaphrynoderma*	CAS247910	Myanmar	KJ128283	KU244449
*Thelodermanebulosum*	ROM 39588	Kon Tum, Vietnam	KT461887	–
*Thelodermalicin*	MVZ 9458	Indonesia	KU244368	KU244447
*Thelodermalateriticum*	VNMN 1216	Bac Giang, Vietnam	LC012851	–
*Thelodermalacustrinum*	NCSM 84683	Vientiane, Laos	KX095246	–
*Thelodermahorridum*	KUHE 52582	Negeri Sembilan, Malaysia	LC012861	–
*Thelodermagordoni*	MVZ 226469	Vinh Phuc, Vietnam	KU244363	KU244451
*Thelodermacorticale*	MVZ 223905	Vinh Phuc, Vietnam	KU244364	KU244452
*Thelodermaauratum*	ZMMU A5828	Gia Lai, Vietnam	MG917767	–
*Thelodermaannae*	NAP05558	Hoa Binh, Vientam	MG917766	–
*Thelodermaasperum*	ZRC1.1.9321	Malaysia	GQ204725	–
*Thelodermabaibungense*	YPX31940	Motuo, Tibet, China	KU981089	–
*Thelodermabicolor*	LC1	Lvchun, Yunnan, China	KY495632	–
*Thelodermamoloch*	GXNU YU000115	Yingjiang, Yunnan, China	MT509809	–
*Thelodermapyaukkya*	GXNU YU000116	Yingjiang, Yunnan, China	MT509810	MT522176
*Thelodermapetilum*	HNUE MNA2012.0001	Dien Bien, Vietnam	KJ802925	–
*Thelodermarhododiscus*	CIB GX200807017	Jinxiu, Guangxi, China	LC012842	–
*Thelodermarhododiscus*	KIZ060821063	Jinxiu, Guangxi, China	EF564533	–
*Thelodermarhododiscus*	KIZ060821170	Jinxiu, Guangxi, China	EF564534	–
*Thelodermarhododiscus*	SCUM 061102L	Jinxiu, Guangxi, China	EU215530	–
*Thelodermarhododiscus*	CIB GX200807048	Jinxiu, Guangxi, China	KJ802921	–
*Thelodermarhododiscus*	GXNU YU000069	Jinxiu, Guangxi, China	OL843957	OL843972
*Thelodermarhododiscus*	GXNU YU000070	Jinxiu, Guangxi, China	OL843958	OL843973
*Thelodermarhododiscus*	GXNU YU000309	Huanjiang, Guangxi, China	OL843959	OL843974
*Thelodermarhododiscus*	GXNU YU000318	Longlin, Guangxi, China	OL843960	OL843975
*Thelodermarhododiscus*	GXNU YU000319	Longlin, Guangxi, China	OL843961	OL843976
*Thelodermarhododiscus*	C051	Jinxiu, Guangxi, China	–	KP996753
*Thelodermarhododiscus*	C089	Jinxiu, Guangxi, China	–	KP996786
*Thelodermarhododiscus*	C090	Jinxiu, Guangxi, China	–	KP996787
*Thelodermahekouense* sp. nov.	GXNU YU000397	Hekou, Yunnan, China	OL843962	OL843977
*Thelodermahekouense* sp. nov.	GXNU YU000398	Hekou, Yunnan, China	OL843963	OL843978
*Thelodermahekouense* sp. nov.	GXNU YU000412	Hekou, Yunnan, China	OL843964	OL843979
*Thelodermahekouense* sp. nov.	GXNU YU000413	Hekou, Yunnan, China	OL843965	OL843980
*Thelodermahekouense* sp. nov.	GXNU YU000495	Hekou, Yunnan, China	OL843966	OL843981
*Thelodermahekouense* sp. nov.	GXNU YU000496	Hekou, Yunnan, China	OL843967	OL843982
*Thelodermahekouense* sp. nov.	AMNH A163893	Vi Xuyen, Ha Giang, Vietnam	DQ283393	–
*Thelodermahekouense* sp. nov.	HHU-WJHK01	Hekou, Yunnan, China	KY495639	–
*Thelodermahekouense* sp. nov.	HHU-WJHK02	Hekou, Yunnan, China	KY495640	–

Sequences were aligned using MUSCLE with the default parameters in MEGA v. 7 (Kumar et al. 2016). Uncorrected pairwise distances between species were calculated in MEGA v. 7. The best substitution model was selected using the Akaike Information Criterion (AIC) in jMODELTEST v. 2.1.10 (Darriba et al. 2012). Bayesian inferences were performed in MRBAYES v. 3.2.6 (Ronquist et al. 2012) under the selected substitution model (GTR + I + G). Two runs were performed simultaneously with four Markov chains starting from random tree. The chains were run for 3,000,000 generations and sampled every 100 generations. The first 25% of the sampled trees were discarded as burn-in after the standard deviation of split frequencies of the two runs was less than a value of 0.01, and then the remaining trees were used to create a consensus tree and to estimate Bayesian posterior probabilities (BPPs).

## ﻿Results

**Figure 2.**
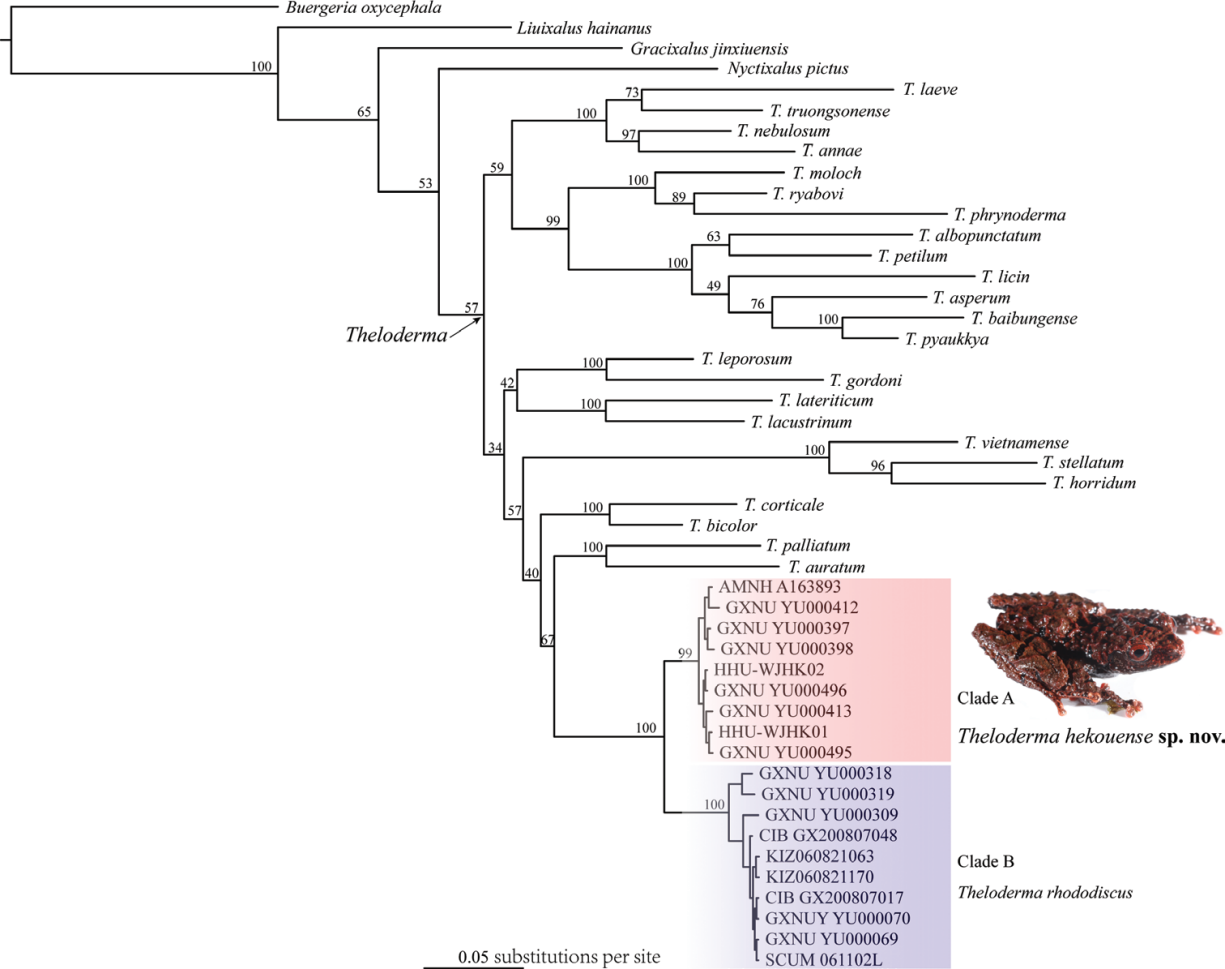
Bayesian phylogram of *Theloderma* inferred from 784 bp of 16S rRNA gene. The values above the branches are Bayesian posterior probabilities.

**Figure 3.**
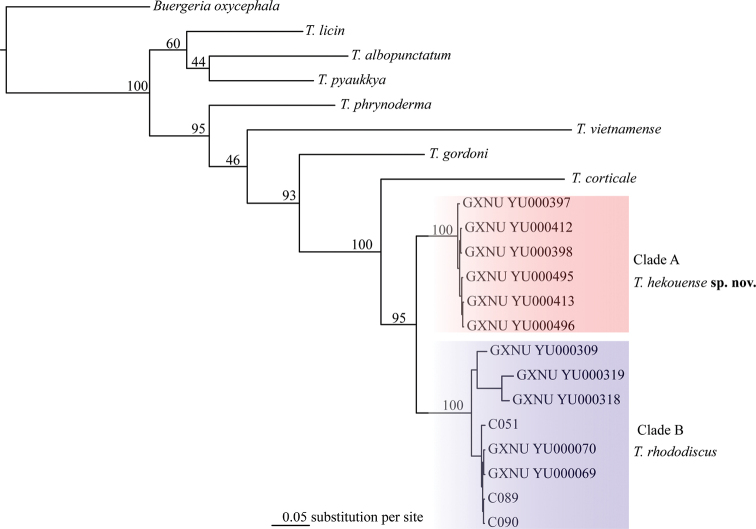
Bayesian phylogram of *Theloderma* inferred from 561 bp of COI gene. The values above the branches are Bayesian posterior probabilities.

The obtained sequence alignments of the 16S and COI genes were 784 bp and 561 bp, respectively. Our phylogenetic analysis strongly supported that specimens from Yunnan and Vietnam form a clade (clade A), which is the sister to the clade consisting of topotypes and other specimens from Guangxi (clade B; Figs 2, 3). The genetic divergence between these two clades is 4.2% and 10.7% in 16S and COI genes, respectively.

The specimens from Hekou, Yunnan, China can be morphologically distinguished from topotypes of *T.rhododiscus* by a series of characters: i.e., red subarticular tubercles, red metacarpal tubercles, a red metatarsal tubercle, and denser white warts on dorsal surface. Therefore, based on the molecular and morphological evidence, we consider the Hekou specimens to represent a cryptic species and describe this species below.

### ﻿ Theloderma hekouense sp. nov.

Taxon classificationAnimaliaAnuraRhacophoridae

041AFD91-E710-5633-8A67-121E18B9BE02

http://zoobank.org/65A68280-DECC-4559-BB78-1FBA7F82474B

Figs 4 , 5A–C

#### Holotype.

GXNU YU000496, adult male, collected on 9 September 2021 by Jian Wang from Hekou, Yunnan, China (22°54'N, 103°42'E, 2109 m a.s.l.; Fig. 1).

#### Paratypes.

GXNU YU000397 and GXNU YU000398, two adult males, collected from the type locality by Jian Wang on 1 May 2021; GXNU YU000413 and GXNU YU000495, two adult males collected from the type locality by Jian Wang on 28 May 2020 and 9 September 2021, respectively; GXNU YU000412, one adult female, collected from the type locality by Jian Wang on 28 May 2020.

#### Etymology.

The specific epithet is named after the type locality, Hekou County, Yunnan, China. We suggested “Hekou Bug-eyed frog” for the common English name and 河口棱皮树蛙 (Hé Kǒu Léng Pí Shù Wā) for the common Chinese name.

#### Diagnosis.

The new species was assigned to genus *Theloderma* by its phylogenetic position and the following morphological characters: distinct tympanum, terminal phalanx with Y-shaped distal end, intercalary cartilage between terminal and penultimate phalanges of digits, tips of digits expanded into large discs bearing circummarginal grooves, head skin not co-ossified to skull (Poyarkov et al. 2018). *Thelodermahekouense* sp. nov. can be distinguished from *T.rhododiscus* and other congeners by having a combination of the following characters: 1) small body size; 2) dorsal surface coarsely rough with large ridges and tubercles; 3) dense warts on dorsal surface; 4) absence of white markings on dorsal surface; 5) iris uniformly reddish brown; 6) discs, metacarpal tubercles, metatarsal tubercles, and subarticular tubercles red; 7) webbing between fingers, vocal sac, and vomerine teeth absent.

#### Description of holotype.

Adult male (SVL 25.7 mm; Table 2); head width (HW 8.5 mm) nearly equal to head length (HL 8.9 mm); snout slopes upward towards the tip, slightly protruding beyond lower jaw in ventral view; canthus rostralis distinct; loreal region sloping; nostrils oval, lateral, nearer tip of snout; interorbital distance (IOD 3.0 mm) greater than internarial distance (IND 2.4 mm) and upper eyelid width (UEW 2.6 mm); pineal spot absent; pupil oval, horizontal; tympanum distinct (TD 2.2 mm), rounded, greater than half eye diameter (ED 3.1 mm); supratympanic fold indistinct; vomerine teeth absent; choanae oval; tongue cordiform, wide deeply notched posteriorly; no vocal sac.

**Table 2.** Measurements (in mm) of *Thelodermahekouense* sp. nov. from the type locality (holotype is marked with asterisk).

Character	GXNU YU000397	GXNU YU000398	GXNU YU000412	GXNU YU000413	GXNU YU000495	GXNU YU000496*
Sex	M	M	F	M	M	M
SVL	25.9	27.2	26.8	25.9	26.2	25.7
HL	8.9	9.0	8.9	8.8	8.9	8.9
HW	8.6	9.0	9.1	8.7	8.5	8.5
SL	3.7	3.8	3.8	3.6	3.6	3.5
IND	2.4	2.5	2.4	2.4	2.3	2.4
IOD	2.9	2.9	3.0	3.0	2.8	3.0
UEW	2.3	2.5	2.7	2.4	2.4	2.6
ED	3.2	3.1	3.3	3.2	3.2	3.1
TD	2.2	2.2	2.3	2.1	2.1	2.2
DNE	2.4	2.5	2.5	2.3	2.2	2.2
FHL	13.7	14.2	14.5	13.5	14.1	13.3
TL	13.9	14.2	14.9	14.0	14.9	13.9
TFL	19.8	20.3	21.2	19.4	20.3	18.9
FL	12.8	13.2	13.9	12.8	13.7	12.3

Forelimbs moderately robust; relative length of fingers I<II<IV<III; all fingertips expanded into discs with circummarginal grooves, relative width of finger disks I<II<IV<III; nuptial pad present on base of finger I; webbing between fingers absent; subarticular tubercles prominent and rounded, formula 1, 1, 2, 2; supernumerary tubercle prominent; two metacarpal tubercles, the outer divided into two.

Hindlimbs long; tibiotarsal articulation reaching tip of snout when hindlimb stretched alongside of body; heels overlapping when legs positioned at right angles to body; tarsal glands absent; relative length of toes I<II<III=V<IV; toe I with preaxial dermal fringe and toe V with postaxial dermal fringe; all toe tips expanded into discs with circummarginal grooves; toes webbed, webbing formula I2-2II1.5–3III2-3IV3-1.75V; subarticular tubercles prominent and rounded, formula 1,1,2,3,2; inner metatarsal tubercle prominent, light red; outer metatarsal tubercle absent.

Dorsolateral fold absent; dorsal surface very rough with prominent irregular ridges, conical tubercles, and dense white small warts on dorsum, top of head, upper eyelids, and dorsal of limbs; head side and body flank rough, scattered with warts; no warts on tympanum; dorsal skin of digits relatively smooth, scattered with white warts; white tubercles and warts around vent; chest, belly, body flank, and ventral surface of forearm and thigh coarsely granular, more so on venter; white tubercles and warts scattered on venter of tarsus and feet.

#### Coloration in life.

Dorsal surface tea-brown with black spots between the nostrils and eyes, between eyes, and on dorsum and dorsal surface of limbs; head side almost uniformly tea-brown, with few white dots on tympanum region; body flank tea-brown, scattered with black spots enclosed by white stripes; a large black spot on sacral area extended to dorsum and connected with the black band on thigh when thigh adhered to body; ventral surface brownish black with white spots on chin and white marbled network on belly and limbs; dorsal and ventral surfaces of discs orange-red; subarticular tubercles, metacarpal tubercles, and metatarsal tubercle semitransparent with light red; nuptial pad greyish white; toe webbing orange-red mottled with dark; iris red-brown.

#### Coloration in preservative.

Dorsal surface faded to brownish black with black spots, pattern as in life; tubercles and warts white; ventral surface brownish black with white spots and white marbled network; discs, subarticular tubercles, metacarpal tubercles, and metatarsal tubercles faded to white (Fig. 5A, B).

#### Morphological variation.

The new species is sexually dimorphic in that the female has no nuptial pad. Black spots on dorsal surface varied among individuals in that 1) GXNU YU000398 and YU000495 have no distinct black spots between snout and eyes, 2) GXNU YU000398 and YU000413 have only one large black spot on dorsum whereas other types have two or more, and 3) GXNU YU000397 has two large black spots between eyes whereas other types have only one.

#### Distribution.

In addition to the type locality, Hekou, Yunnan, China, the new species also occurs in Ha Giang, northern Vietnam (Bain and Nguyen 2004) because our molecular analyses revealed that the samples from Ha Giang also belong to the clade of the new species. The new species inhabits shrubs and prefers to breed in water-filled tree hollows. All specimens from Yunnan were found in an artificial breeding trap constructed using water bottles for surveillance of amphibian diversity (Fig. 6).

#### Comparisons.

Orlov et al. (2006) identified three groups in *Theloderma* based on SVL, including small (28–35 mm), medium-sized (40–45 mm), and large (48–75 mm). Here the new species (adult SVL 25.7–27.2 mm) is referred to the small group, and therefore can be easily distinguished from members of the other two groups including: *T.bicolor*, *T.corticale* (male SVL 61 mm, *n* = 1), *T.gordoni* (male SVL 36.4–46.7 mm), *T.horridum* (SVL 37.1–48.7 mm, *n* = 4), *T.leporosum* (SVL 62.6 mm, *n* = 1), *T.moloch* (SVL 39.6–46.3 mm in two females and SVL 40.2 mm in one male), *T.nagalandense* (male SVL 52.8 mm, *n* = 1), *T.phrynoderma* (SVL 41.4–44.6 mm), and *T.ryabovi* (male SVL 43.8 mm, *n* = 1).

**Figure 4.**
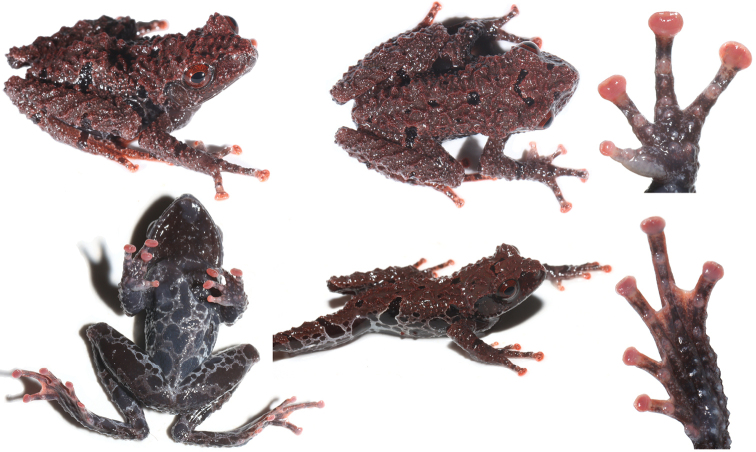
Views of holotype of *Thelodermahekouense* sp. nov. (GXNU YU000496) in life.

A morphological comparison between small-bodied *Theloderma* species is summarized in Table 3. The new species can be distinguished from its sister-species *T.rhododiscus*, with which it was previously confused, by the denser white warts on dorsal surface (vs relatively sparse), red subarticular tubercles (vs white), red metacarpal tubercles (vs white), a red metatarsal tubercle (vs white), and dorsal and ventral surfaces blackish in preservative (vs tea-brown) (Fig. 4).

**Table 3.** Morphological comparison of members of *Theloderma* with small size (SVL < 35 mm). “?” means unknown.

Species	Iris color	Finger webbing	Color of discs	Dorsal colour	Ventral colour	Vomerine teeth	Vocal sac	Dorsal skin	Metacarpal, metatarsal, and subarticular tubercles
*T.hekouense* sp. nov.	red brown	absent	both dorsal and ventral surfaces orange red	tea-brown with no white markings	brownish black with white marbled network	absent	absent	coarsely rough with large asperities	red
*T.annae*	greyish green	absent	both dorsal and ventral surfaces greyish white	greyish green	greyish white	absent	absent	smooth	gray
*T.albopunctatum*	red brown	present	both dorsal and ventral surfaces brown	brown with white markings	dark olive with white stripes	absent	present	smooth with small asperities	greyish white
*T.asperum*	reddish brown	absent	both dorsal and ventral surfaces brown	dark grey-brown with white markings	marbled black and bluish grey/white	absent	present	rough with large asperities	?
*T.auratum*	golden above and black below	absent	dorsal surface dark brown and ventral surface grey	golden yellow	greyish blue with brown blotches	absent	absent	smooth	gray
*T.baibungense*	red brown	absent	dorsal surface black brown and ventral surface grey	brown with white markings	black with white stripes	absent	present	smooth with small asperities	white
*T.lacustrinum*	uniformly bronze	absent	dorsal and ventral surfaces bronze	light brown	uniformly gray	absent	?	smooth with small asperities	gray
*T.lateriticum*	deep brick-red	absent	both dorsal and ventral surfaces grey	brick-red	grey-brown with white spots	absent	absent	granular with small bumps	gray-brown
*T.laeve*	grey above and dark brown below	absent	both dorsal and ventral surfaces grey	beige with thin light middorsal stripe	uniformly violet-grey	absent	absent	smooth	grey
*T.licin*	red	present	dorsal surface black-brown	pale whitish brown to light brown	white with brown reticulation	absent	present	nearly smooth with fine asperities	?
*T.nebulosum*	pale gold above and reddish brown below	absent	both dorsal and ventral surfaces brown	brown with dark patterning	dark brownish black with pale blue/white marbling	absent	?	nearly smooth with very sparsely distributed minute asperities	brown
*T.palliatum*	pale gold above and dark red below	absent	both dorsal and ventral surface brown to greyish brown	pale to medium brown with dark brown blotches	dark warm brown with pale bluish white marbling	absent	absent	weakly rugose with sparsely scattered minute asperities	faint white
*T.petilum*	reddish brown above and grey below	absent	dorsal surface lavender and ventral surface creamy-white	light brown with dark brown reticulations	creamy white	present	?	nearly smooth with small, white asperities	creamy white
*T.pyaukkya*	uniformly red	absent	dorsally red and ventrally brown	brown with white markings	brown with cream marbling	absent	present	rough with fine asperities	grayish white
*T.rhododiscus*	uniformly red-brown	absent	both dorsal and ventral surface red	tea-brown with black blotches	brownish black with gray-white network	absent	absent	rough with large asperities	white
*T.stellatum*	dark gold with black	present	dorsal surface reddish and ventral surface grey	brown with white markings	cream with purplish-brown flecks or spots	absent	absent	rough with small or large asperities	flesh-white
*T.truongsonense*	golden yellow above and black below	absent	dorsal surface beige to black brown and ventral surface	yellow-goldish with dark brown	dark gray with black speckles	absent	?	smooth with small asperities	gray
*T.vietnamense*	golden-brownish	present	dorsally reddish and ventrally grey	brown with white markings	dark brown to blackish with slight whitish to bluish reticulations	absent	present	rough with large ridges and warts	whitish to bluish

**Figure 5.**
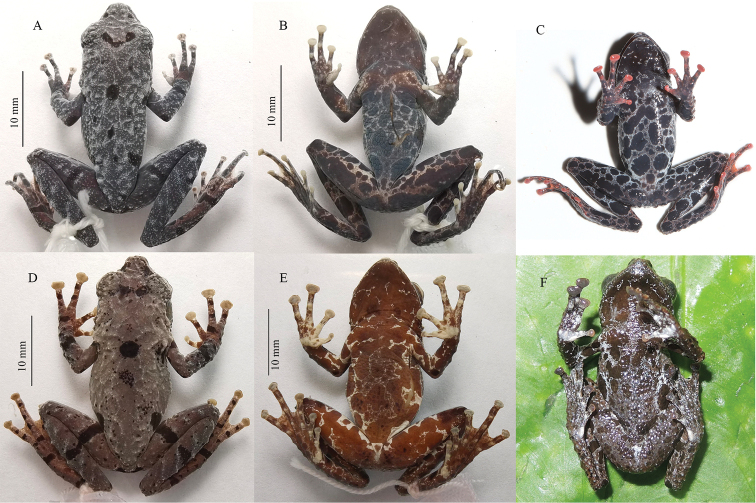
*Thelodermahekouense* sp. nov. and *T.rhododiscus***A–C** dorsal and ventral views of *Thelodermahekouense* sp. nov. **A, B** holotype (GXNU YU000496) in preservative **C** paratype (GXNU YU000495) in life **D–F***T.rhododiscus***D, E** topotype (GXNU YU000069) in preservative **F** topotype (GXNU YU000417) in life.

**Figure 6.**
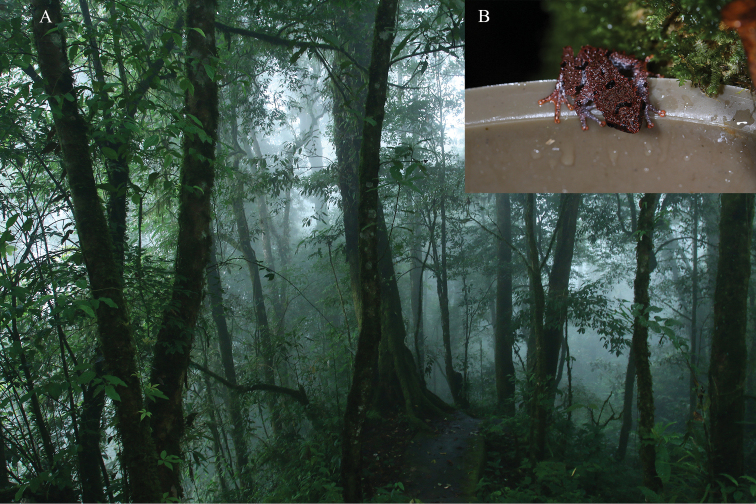
Habitat of *Thelodermahekouense* sp. nov. **A** habitat at the type locality **B** an individual found in a water bucket that was set up in the field as potential breeding site of treefrog preferred breeding in water-filled tree holes by the authors for amphibian monitoring.

*Thelodermahekouense* sp. nov. is distinguishable from *T.annae*, *T.auratum*, *T.laeve*, *T.lacustrinum*, *T.lateriticum*, *T.licin*, *T.nebulosm*, *T.palliatum*, *T.petilum*, and *T.truongsonense* by having the dorsal surface coarsely roughened with large ridges and tubercles (vs smooth or weakly rugose with small asperities), and from *T.albopunctatum*, *T.asperum*, *T.baibungense*, *T.pyaukkya*, *T.stellatum*, and *T.vietnamense* by absence of white markings on the dorsal surface (vs present).

The new species further differs from *T.annae*, *T.auratum*, *T.lacustrinum*, *T.laeve*, *T.nebulosm*, *T.palliatum*, *T.petilum*, *T.stellatum*, *T.truongsonense*, and *T.vietnamense* by the uniformly reddish-brown iris (vs lacking red colouration or bicoloured); from *T.albopunctatum*, *T.licin*, *T.stellatum*, and *T.vietnamense* by lacking webbing between the fingers (vs present); from *T.albopunctatum*, *T.asperum*, *T.baibungense*, *T.licin*, *T.pyaukkya*, and *T.vietnamense* by lacking a vocal sac (vs present); from *T.petilum* by lacking vomerine teeth (vs present); from *T.annae*, *T.albopunctatum*, *T.asperum*, *T.auratum*, *T.baibungense*, *T.lacustrinum*, *T.lateriticum*, *T.laeve*, *T.licin*, *T.nebulosum*, *T.palliatum*, *T.petilum*, *T.pyaukkya*, *T.stellatum*, *T.truongsonense*, and *T.vietnamense* by having both dorsal and ventral surfaces of the discs reddish brown (vs lacking red colouration or red only on the dorsal surface); and from all small-bodied congeners in having red metacarpal, metatarsal, and subarticular tubercles (vs lacking red colouration).

## ﻿Discussion

*Thelodermarhododiscus* was thought to have a broad distribution ranging from eastern China to southwestern China and northern Vietnam (Zeng et al. 2017). Although previous molecular studies have revealed relatively large genetic divergence between samples from the type locality and limited samples from Yunnan and Vietnam (e.g., Poyarkov et al. 2015; Hou et al. 2017b), the taxonomic status of *T.rhododiscus* from the western part of its distribution (Yunnan and Vietnam) has never been doubted in previous publications. In this study, our molecular data and morphological comparison supports that the taxon known as *T.rhododiscus* from Yunnan, China and adjacent northern Vietnam should be considered representing a sibling species of *T.rhododiscus*, from which the new species differs morphologically by denser white warts on the dorsal surface and red subarticular, metacarpal, and metatarsal tubercles, and genetically by 4.2% and 10.7% divergence in 16S rRNA and COI genes, respectively.

With the exclusion of Yunnan and northern Vietnam from the geographic range of *T.rhododiscus*, the range of *T.rhododiscus* should be revised to include Guangxi, Guangdong, Hunan, Fujian, and Jiangxi. In Guangxi, *T.rhododiscus* was previously known from three areas including Jinxiu (Dayao Mt National Natural Reserve), Longsheng (Huaping National Natural Reserve), and Nanning (Daming Mt National Natural Reserve) (Zeng et al. 2017). In this study, we found two new occurrences of *T.rhododiscus* in northern and northwestern Guangxi, including Longlin and Huanjiang counties. The former is adjacent to southwestern Guizhou and eastern Yunnan and the latter is adjacent to southern Guizhou. Therefore, it can be expected that *T.rhododiscus* will be found from Guizhou and eastern Yunnan in the future.

Yunnan is the region richest in species of bug-eyed frogs in China. With the addition of *T.hekouense* sp. nov., there are now 10 *Theloderma* species in China and seven of them are distributed in Yunnan including *T.albopunctatum*, *T.baibungense*, *T.bicolor*, *T.gordoni*, *T.moloch*, *T.pyaukkya*, and *T.hekouense* sp. nov. Most of these species were recorded from there recently (e.g., Hou et al. 2017b; Qi et al. 2018; Du et al. 2020), indicating that species diversity of *Theloderma* in Yunnan was obviously underestimated probably owing to that *Theloderma* species are not easy to be found because of their preference of breeding in water-filled tree hollows. Taxonomic progress of amphibians from Yunnan in recent years (e.g., Yuan et al. 2018; Yu et al. 2019a, 2019b; Du et al. 2020; Jiang et al. 2020) reflects that amphibian diversity in Yunnan remains to be poorly known. Beside *T.rhododiscus* mentioned above, we expect that more *Theloderma* species known from adjacent regions will be found from southern Yunnan, China (e.g., *T.corticale*, *T.lateriticum*, and *T.petilum*).

## Supplementary Material

XML Treatment for Theloderma hekouense
